# Molecular Prevalence and Subtypes Distribution of *Blastocystis* spp. in Humans of Latin America: A Systematic Review

**DOI:** 10.3390/tropicalmed9020038

**Published:** 2024-02-01

**Authors:** Carmine Fusaro, Jaime E. Bernal, Rosa Baldiris-Ávila, Rafael González-Cuello, Julio Cisneros-Lorduy, Arley Reales-Ruiz, Raimundo Castro-Orozco, Yohanna Sarria-Guzmán

**Affiliations:** 1 Facultad de Ingenierías, Universidad de San Buenaventura, Cartagena de Indias, Bolivar 130010, Colombia; carmine.fusaro@usbctg.edu.co (C.F.); julio.cisneros@usbctg.edu.co (J.C.-L.); aarealesr@miusbctg.edu.co (A.R.-R.); 2 Facultad de Medicina, Universidad del Sinú, Cartagena de Indias, Bolivar 130001, Colombia; jebernal@gmail.com; 3 Facultad de Ciencias Exactas y Naturales, Universidad de Cartagena, Cartagena de Indias, Bolivar 13001, Colombia; rbaldirisa@unicartagena.edu.co; 4 Facultad de Ingeniería, Universidad de Cartagena, Cartagena de Indias, Bolivar 130015, Colombia; rgonzalezc1@unicartagena.edu.co; 5 Facultad de Ciencias de la Salud, Universidad de San Buenaventura, Cartagena de Indias, Bolivar 130010, Colombia; rcastro@usbctg.edu.co

**Keywords:** *Blastocystis*, Latin America, molecular, humans, systematic review

## Abstract

*Blastocystis* spp. are among the few enteric parasites with a prevalence that can reach up to approximately 80% in communities of developing countries. This systematic review updates and summarizes available literature on the molecular prevalence and subtype distribution of *Blastocystis* spp. in Latin American people. This work follows the PRISMA (Preferred Reporting Items for Systematic Reviews and Meta-Analyses) guidelines. The literature revised covers from 1 January 2015 to 6 October 2023 in seven different scientific databases, and the material was selected through inclusion and exclusion criteria. According to data found in the 36 selected articles, the prevalence of *Blastocystis* spp. in Latin America ranged between 5.8% (Bolivian rural communities) and 94.0% (Colombian general public). Generally, genomic DNA was extracted from approximately 200 mg fecal sediments using commercial kits, such as the QIAamp Stool Mini Kit (QIAGEN, Hilden, Germany) or the Norgen Stool DNA Isolation Kit (Norgen Biotek Corporation, Thorold, ON, Canada). Subtype-specific primers (such as the couple of primers BhRDr–RD5) developed from unique sequences of the SSU rRNA gene were applied to *Blastocystis* subtyping. Ten specific subtypes (STs) were found as well as various mixed infections, and the most circulating *Blastocystis* STs were in the order ST3, ST1, ST2, and ST4. The most recent data about *Blastocystis* spp. molecular epidemiology and the STs in communities of Latin America are limited to studies from specific countries. Novel scientific data from the other countries are required to obtain a complete picture and truly understand the distribution and prevalence of *Blastocystis* spp. and the STs.

## 1. Introduction

*Blastocystis* spp. are anaerobic, unicellular, intestinal parasitic protists distributed worldwide and able to colonize the large intestine of many vertebrate species, including humans [1,2,3,4]. *Blastocystis* spp. present multiple evolutionary stages or life cycles, i.e., vacuolar, granular, multi-vacuolar, a-vacuolar, ameboid, and cystic forms [5,6].

The main methods to detect *Blastocystis* spp. are microscopy, culturing, and molecular assays [7]. Two standard sets of primers, i.e., BhRDr/RD5 (~600 bp, Barcoding primers) and Blast 505–532/Blast 998–1017 (~500 bp, Santin primers), were frequently used for sequence regions of the small subunit ribosomal RNA (*SSU rRNA*) gene and typified *Blastocystis* spp. [8,9]. Molecular methods include: (1) restricted fragment length polymorphism (RFLP), (2) subtype-specific sequence-tagged site (STS), and (3) real-time polymerase chain reaction (PCR) [10,11]. Maloney, Molokin, and Santin [12], more recently, developed a novel method using Oxford Nanopore MinION long-read sequencing and universal eukaryotic primers to produce full-length (>1800 bp) SSU rRNA gene sequences for *Blastocystis* spp. The Public Databases for Molecular Typing and Microbial Genome Diversity (PubMLST) reports provenance and phenotype information linked to *Blastocystis* spp. molecular typing information [13].

*Blastocystis* spp., isolated from both human and animal hosts, are currently classified into 18S rRNA gene subtypes (STs), i.e., ST1–ST17, ST21, and ST23–ST32 [13,14]. Many STs, i.e., ST1–ST8, ST10, ST12, ST14, and ST16, are detected in different hosts (humans and domestic and wild animals) [13,15,16]. The frequency and variety of *Blastocystis* spp. STs in various species of animals strengthens the hypothesis of parasite zoonotic transmission [17,18]. Four STs, i.e., ST1, ST2, ST3, and ST4, are found frequently in human studies [19,20,21,22]; to date, ST9 has been identified only in humans [16,18]. However, complete SSU rRNA gene sequences are available for 17 STs (ST1–ST17) [23,24].

*Blastocystis* spp. are among the few enteric parasites with a prevalence that frequently exceeds 5% in citizens of industrialized countries [25] and can reach up to approximately 80% in developing countries [26,27]. The parasite transmission among humans generally occurs via the fecal–oral route, and may be either direct (i.e., person-to-person or zoonotic) or indirect (i.e., foodborne and waterborne) [28,29].

People living in rural contexts or developing countries, such as those located in the Caribbean and Latin America, with deficient water sanitary supply services, inadequate wastewater treatments, and close animal contact, are particularly exposed to health risks derived from a *Blastocystis* spp. infection [30]. Additionally, favorable climatic conditions, i.e., high humidity and warm temperature, increase the chance of transmission of *Blastocystis* spp. in tropical areas [31,32]. The *Blastocystis* spp.-infected patients are, in many cases, asymptomatic or generally experience mild symptoms, such as diarrhea, abdominal pain, flatulence, bloating, constipation, and skin lesions [33]. However, specific groups of individuals, i.e., children, elderly people, and patients with anemia or irritable bowel syndrome, could experiment serious Blastocystosis symptoms [16,34,35].

The occurrence of *Blastocystis* spp. has been observed in various water sources [36,37], including rivers [38], lakes [39], streams [40], sewage [41], surface water [42], stored water [43], tap water [44], and water tanks [45]. Outbreaks of Blastocystosis in poor communities have been linked to drinking water contaminated with fecal matter and poor sanitation services [40,46]. Scientific reports and evidence about the occurrence and prevalence of parasites in water sources have given the international community a boost to adopt safety strategies, such as the safe drinking water, sanitation, and hygiene programs (WASH) of the World Health Organization (WHO) [47].

Despite the recent efforts of the scientific community, governments, and international health organizations to detect and typify *Blastocystis* spp. in the Caribbean and Latin American countries, the epidemiological–molecular characterization and spatial distribution are not yet fully clarified. This systematic review updates and summarizes available literature on the molecular prevalence and subtype distribution of *Blastocystis* spp. in individuals of Latin America.

## 2. Materials and Methods

The systematic review was performed according to the guidelines set forth in the Preferred Reporting Items for Systematic Reviews and Meta-Analyses (PRISMA) and the checklist of Moher et al. [48] (Supplementary File S1).

### 2.1. Search Strategy

Searching the literature was carried out on 6–7 October 2023 by an author (Y.S.-G.). Full-text articles were searched in seven electronic scientific databases, including ISI Web of Science (Clarivate Analytics), EMBASE (Elsevier), Science Direct (Elsevier), Scopus (Elsevier), SciELO (São Paulo Research Foundation—FAPESP), PubMed (National Library of Medicine of USA—NLM), and EBSCOhost (EBSCO Industries), using the following Boolean equation: (“*Blastocystis*”) AND (“Argentina” OR “Belize” OR “Bolivia” OR “Brazil” OR “Chile” OR “Colombia” OR “Costa Rica” OR “Cuba” OR “Ecuador” OR “El Salvador” OR “Guatemala” OR “Guyana” OR “French Guyana” OR “Honduras” OR “Mexico” OR “Nicaragua” OR “Panama” OR “Paraguay” OR “Peru” OR “Puerto Rico” OR “Dominican Republic” OR “Suriname” OR “Uruguay” OR “Venezuela”).

### 2.2. Inclusion Criteria

The inclusion criteria, applied to full texts for assessing their eligibility, were: (a) original article focusing on molecular identification of *Blastocystis* spp. in Latin American humans, (b) article published from 1 January 2015 to 6 October 2023, (c) article written in English and/or Spanish, (d) study limited to humans, (e) cross-sectional study, and (f) article published in peer-reviewed journals inserted in the Scimago Journal Ranking (SJR) database.

### 2.3. Exclusion Criteria

The exclusion criteria, applied to full texts for assessing their eligibility, were: (a) abstract not associated with the full article, (b) article published in non-peer-reviewed source, (c) article not written in English or Spanish, (d) review papers of literature or meta-analyses, (e) retrospective studies, (f) short communication, (g) letters to the editor, and (h) studies with ≤3 points based on the Joanna Briggs Institute (JBI) tool [49].

### 2.4. Selection of Studies

The Mendeley Desktop Reference Management System 1.19.8 software was used to compile the identified articles and to remove the duplicates. Subsequently, articles were independently screened for title and abstract pertinence by two authors (Y.S.-G. and J.E.B.). Irrelevant titles were removed. Disagreements between the two researchers were resolved through consultation with a third author (C.F.). Inclusion and exclusion criteria were applied to full texts to assess their eligibility. Two authors (Y.S.-G. and J.E.B.) independently analyzed the full-text papers and only those that met all criteria were finally selected.

### 2.5. Data Extraction and Analysis

The information from each selected paper was extracted and organized in a matrix with the following subjects: (a) reference, (b) quartile, (c) rural/urban, (d) quality, (e) collection period, (f) demographic group studied, (g) age, (h) number of repeat samples, (i) concentration method, (j) DNA extraction method, (k) *Blastocystis*-specific SSU-rDNA primers, (l) product size, (m) amplification, (n) subtypes, (o) prevalence, and (p) 95% confidence intervals (CI).

### 2.6. Quality Assessment

The quality of the included studies was assessed with standardized critical appraisal instruments from the JBI for prevalence [49]; the tool, composed by nine questions with four answer options (yes, no, unclear, and not applicable), assists in assessing the trustworthiness, relevance, and results of published scientific articles.

Based on a quality score rating system, the papers were divided into two categories: high-quality study (score 7–9) and moderate-quality study (score 4–6).

The nine questions (Q) of the JBI instrument are: (Q1) Was the sample frame appropriate to address the target population? (Q2) Were study participants sampled in an appropriate way? (Q3) Was the sample size adequate? (Q4) Were the study subjects and the setting described in detail? (Q5) Was the data analysis conducted with sufficient coverage of the identified sample? (Q6) Were valid methods used for the identification of the condition? (Q7) Was the condition measured in a standard, reliable way for all participants? (Q8) Was there appropriate statistical analysis? (Q9) Was the response rate adequate and, if not, was the low response rate managed appropriately?

Two researchers (Y.S.-G. and J.E.B.) worked independently, and disagreements were resolved through consultation with a third author (C.F.) (Supplementary File S2).

## 3. Results

### 3.1. Literature Search

**Figure 1:**
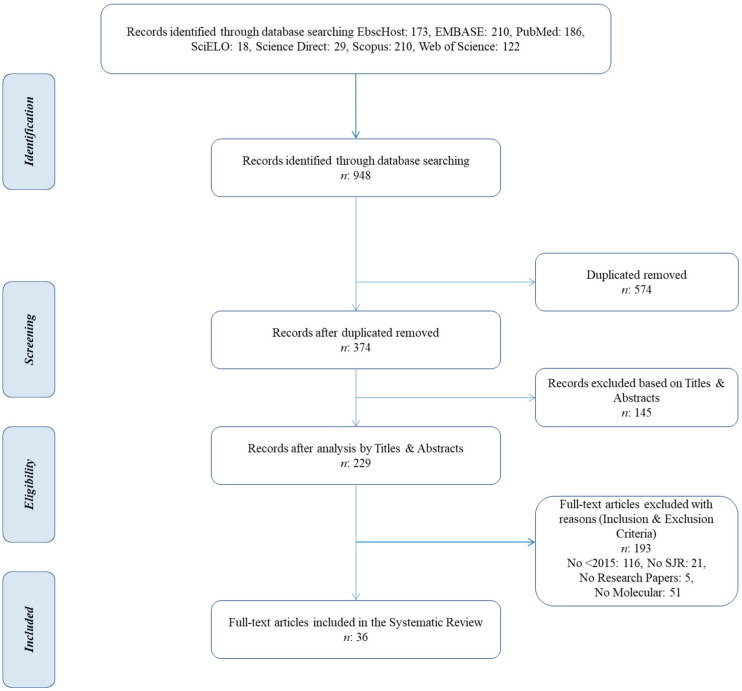
PRISMA flow diagram.

The PRISMA Statement flow diagram that indicates the four phases of the literature search (identification, screening, eligibility, and inclusion) is shown in Figure 1. During the identification phase, a total of 948 publications were recorded. Duplicated articles were automatically removed via a bibliographic management software and the remaining 374 papers were screened for title and abstract pertinence.

The eligibility of 229 full-text articles that passed the title and abstract screening phase was assessed based on preset inclusion and exclusion criteria. Finally, 36 articles [50,51,52,53,54,55,56,57,58,59,60,61,62,63,64,65,66,67,68,69,70,71,72,73,74,75,76,77,78,79,80,81,82,83,84,85] were included in this systematic review.

### 3.2. Characteristics of Included Studies

**Figure 2:**
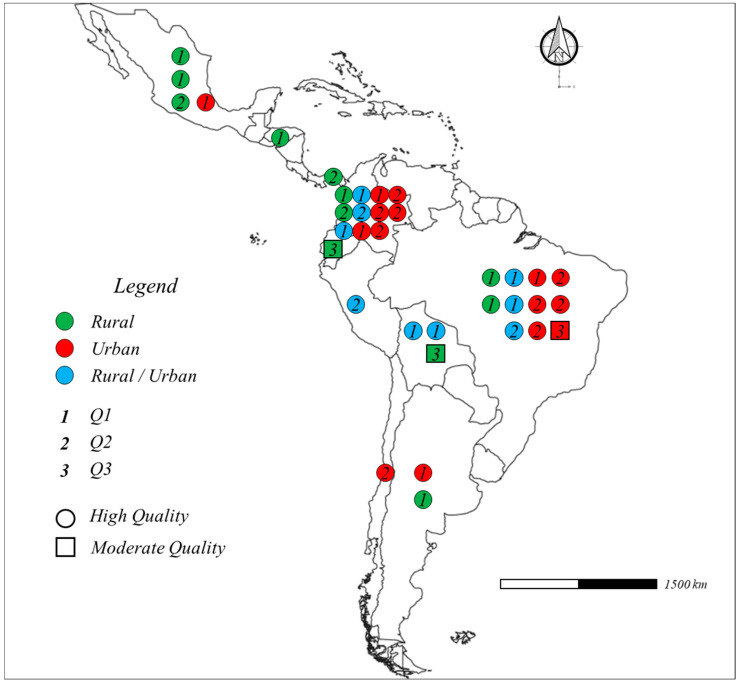
General characteristics of the included studies [50,51,52,53,54,55,56,57,58,59,60,61,62,63,64,65,66,67,68,69,70,71,72,73,74,75,76,77,78,79,80,81,82,83,84,85].

Figure 2 shows the eighteen articles published in Q1 SJR journals [50,51,52,54,55,61,63,64,65,70,71,75,76,77,79,80,81,82], the fifteen articles in Q2 SJR journals [57,58,59,60,62,66,67,68,69,72,73,74,83,84,85], and the three articles found in Q3 SJR journals [53,56,78].

Based on the JBI score rating system tool, thirty-three articles were considered as high-quality scientific papers [50,51,52,54,55,57,58,59,60,61,62,63,64,65,66,67,68,69,70,71,72,73,74,75,76,77,79,80,81,82,83,84,85] and only three were of moderate quality [53,56,78]. The studies were reported from Argentina [50,51], Bolivia [52,53,54], Brazil [55,56,57,58,59,60,61,62,63,64,65], Chile [66], Colombia [67,68,69,70,71,72,73,74,75,76,77], Ecuador [78], Honduras [79], Mexico [80,81,82,83], Panama [84], and Peru [85]. Fifteen studies were conducted on urban citizens [51,56,57,58,59,62,64,66,67,69,71,73,74,75,80], twelve were performed in rural settings [50,53,61,65,68,77,78,79,81,82,83,84], and nine studies focused on rural–urban mixed communities [52,54,55,60,63,70,72,76,85]. The papers, mainly cross-sectional studies, investigated the prevalence and typification of *Blastocystis* spp. in different Latin American social groups, i.e., aborigines, children, teenagers, pregnant women, farmers, urticaria patients, diabetes mellitus patients, other patients, and the elderly [50,51,52,53,54,55,56,57,58,59,60,61,62,63,64,65,66,67,68,69,70,71,72,73,74,75,76,77,78,79,80,81,82,83,84,85] (Table 1).

### 3.3. Molecular Characteristics of the Selected Articles

The main characteristics of the applied experimental methodologies are shown in Table 2.

The most common concentration methods, applied to separate protozoan cysts from excess fecal debris and to detect scanty microorganisms, were Ritchie methods [50,56,60,63,67,70,71,74,80], Kato–Katz [52,63,71,74,79], the zinc sulfate flotation technique [57,59,65,73], and formalin-ethyl acetate concentration techniques [51,75,84]. Other methods, such as spontaneous sedimentation [58,61,64] and centrifugal sedimentation [55], were routinely used. Eight studies did not report the concentration method used [62,72,76,77,78,81,82,83].

In general, genomic DNA was extracted from approximately 200 mg fecal sediments using commercial kits, such as the QIAamp Stool Mini Kit (QIAGEN, Hilden, Germany) [50,51,55,57,58,59,60,61,62,63,64,65,66,68,69,70,78,81,82,83,84] or the Norgen Stool DNA Isolation Kit (Norgen Biotek Corporation, Thorold, ON, Canada) [67,71,72,74,75,77,85], following the manufacturer’s instructions. Other commercial kit, such as the MP FastDNA soil kit [76,79], NucleoSpin Tissue kit [53,54], and Magnex DNA kit [56], were also used. Only Potes-Morales et al. [73] achieved rapid isolation and purification of genomic DNA through the phenol-chloroform isoamyl alcohol extraction method.

Subtype-specific primers developed from unique sequences of the SSU rRNA gene were applied to *Blastocystis* subtyping. For instance, the BhRDr primer was designed to be combined with the RD5 primer, and the expected size of the PCR product is 600 bp. This set of primers was used in 21 of the 36 selected studies [50,51,52,53,54,55,56,57,58,59,60,61,62,63,64,65,66,67,68,69,70,71,72,73,74,75,76,77,78,79,80,81,82,83,84,85]. Other sets of primers, such as F1/R1 (1100 bp SSU rRNA, barcoding region) [53,54,56,80] and Blast 505–532/Blast 998–1017 (500 bp SSU rRNA, barcoding region) [61,64,68,81], were used in just a few studies.

Different molecular approaches, i.e., PCR developed in 29 studies [50,51,52,53,54,55,57,58,59,60,61,62,63,64,65,66,67,68,70,71,72,73,74,78,80,81,83,84,85], qPCR [75,76,77,82], PCR-RFLP [56], multi-parallel qPCR [82], and semi/nested PCR [69], were used to amplify small segments of DNA.

### 3.4. Prevalence of Blastocystis spp. and Subtypes

**Figure 3:**
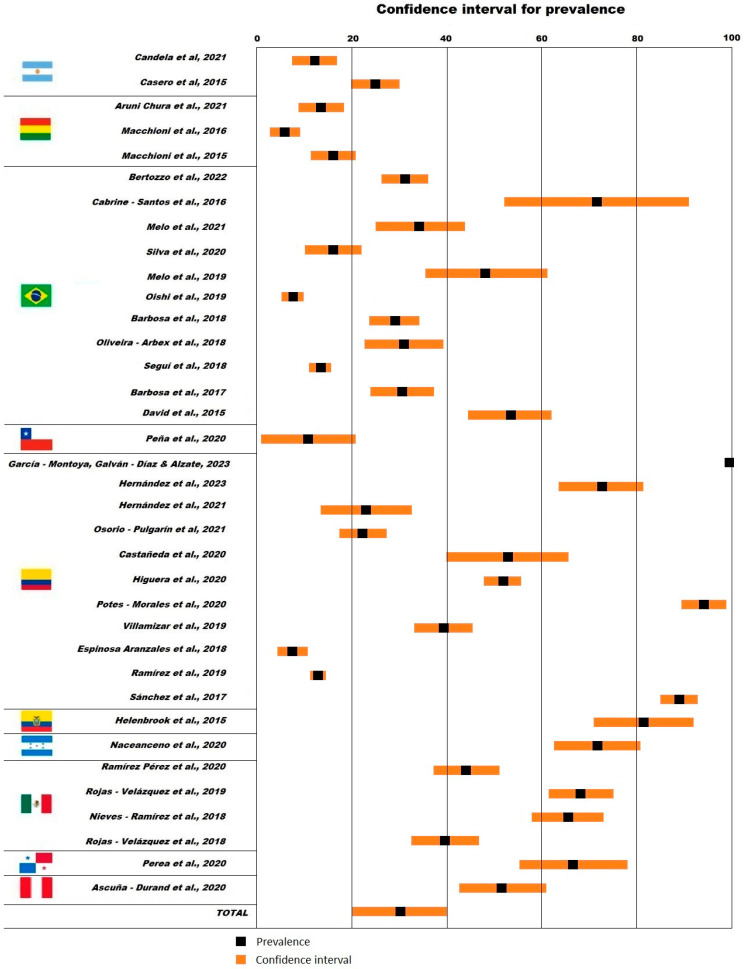
Reported prevalence of *Blastocystis* spp. in Latin America [50,51,52,53,54,55,56,57,58,59,60,61,62,63,64,65,66,67,68,69,70,71,72,73,74,75,76,77,78,79,80,81,82,83,84,85].

The size of the studied groups ranged between 21 [56] and 2026 individuals [76]. The prevalence of *Blastocystis* spp. (Figure 3) in the analyzed fecal samples based on molecular identification methods ranged between 5.8% (Bolivian rural communities, confidence interval (CI) 2.8–8.9%) [53] and 94.0% (Colombian general public, CI 89.3–98.7%) [73]. One-third of the selected studies reported a prevalence of *Blastocystis* spp. less than 20.0% in specific population groups of Argentina [50], Bolivia [52,53,54], Brazil [58,60,63], Chile [66], and Colombia [75,76]. Only three studies reported a prevalence of *Blastocystis* spp. higher than 80.0% in Ecuadorian and Colombian people [73,77,78].

**Figure 4:**
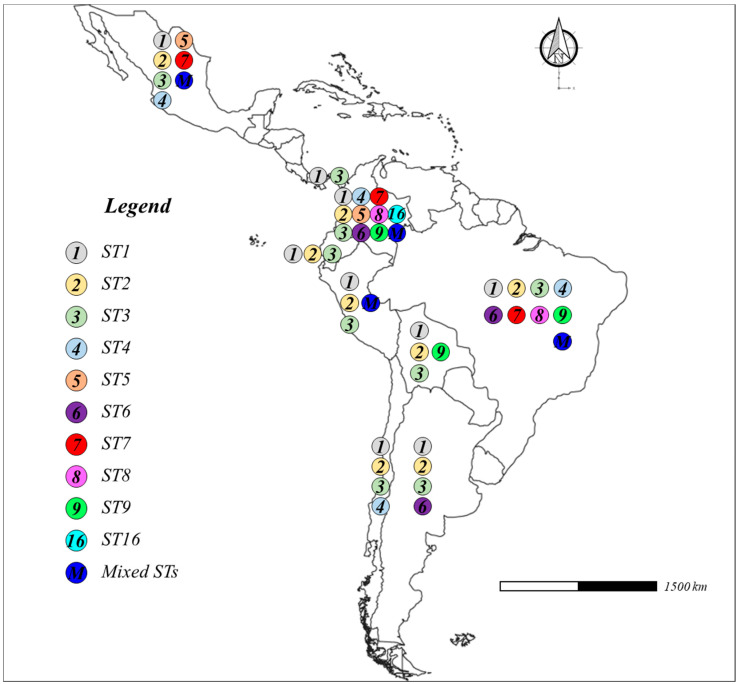
*Blastocystis* subtypes (STs) in Latin America [50,51,52,53,54,55,56,57,58,59,60,61,62,63,64,65,66,67,68,69,70,71,72,73,74,75,76,77,78,79,80,81,82,83,84,85].

Ten STs were found, as follows: ST1 [50,51,52,53,55,56,57,58,60,61,62,63,64,65,66,67,68,69,70,72,73,74,76,77,78,80,81,84,85], ST2 [50,51,52,53,54,55,56,57,58,59,60,61,62,63,64,65,66,67,68,69,70,72,73,74,76,77,78,80,81,85], ST3 [50,51,52,53,54,55,56,57,58,59,60,61,62,63,64,65,66,67,68,69,70,72,73,74,76,77,78,80,81,82,83,84,85], ST4 [55,59,61,63,64,66,68,70,72,74,76,77,80], ST5 [68,80], ST6 [51,55,57,59,63,65,69,70,76,77], ST7 [55,57,58,62,65,76,80], ST8 [57,61,63,72], ST9 [54,55,72], and ST16 [70]. The first three STs were widely distributed in Latin America, while ST16 was only identified by Osorio-Pulgarin et al. [70]. Finally, mixed STs were reported in few study cases, for example, ST1 + ST2 [68,81], ST1 + ST3 [59,60,64,65,67,81,85], ST1 + ST2 + ST3 [68,85], ST2 + ST3 + ST4 [68], ST2 + ST3 [81], ST2 + ST5 [68], and ST3 + ST7 [65]. The STs found in the selected articles are reported in Figure 4.

## 4. Discussion

### 4.1. Epidemiology of Blastocystis spp. in Latin America

*Blastocystis* spp. comprise a genus of single-celled parasites that present a cosmopolitan distribution and colonize an estimated 1 to 2 billion people worldwide, many of them living in developing countries [19]. Epidemiological studies at a molecular level have clearly demonstrated that humans can be colonized by one or more *Blastocystis* STs, some of which are commonly found in non-human hosts [71].

Our findings suggest that only a few Latin American countries report *Blastocystis* spp. molecular data and parasite prevalence rates in their demographic groups. Most of the selected studies were carried out in Brazil [55,56,57,58,59,60,61,62,63,64,65], Colombia [67,68,69,70,71,72,73,74,75,76,77], and Mexico [80,81,82,83].

Brazil is a megadiverse country that possesses six terrestrial biomes and many regional differences in social, economic, and cultural characteristics [86]. A large percentage of municipalities are not equipped with efficient sewage treatment systems and waste management sites [87]. These structural gaps can amplify the diffusion of tropical neglected diseases, including those caused by many *Blastocystis* STs, especially in the poor and vulnerable demographic groups [88]. The *Blastocystis* STs identified in Brazil were ST1–ST4 and ST6–ST9, with the most prevalent being ST1, ST2, and ST3 [55,56,57,58,61,62,63]; furthermore, some data indicate mixed infections, such as ST1 + ST3 [59,60,64,65]. The prevalence rate varied from 7.5% (patients—laboratory) [60] to 71.4% (schoolchildren) [56]. Various studies about the prevalence of *Blastocystis* spp. in many Brazilian contexts were performed in the last eight years. Barbosa et al. [61] indicated an overall prevalence of intestinal parasitic infections above 50.0% and a wide range of *Blastocystis* spp. STs (ST1, ST2, ST3, ST4, and ST8) among the people who belong to a rural community in Rio de Janeiro. *Blastocystis* spp. STs, isolated by Barbosa et al. [64] in a carioca urban community, were genetically highly divergent, with ST3 being the most common among the participants, followed by ST1, ST2, and ST4. A mixed infection (ST1 + ST3) was detected in a few cases. David et al. [65] revealed large genetic variation of *Blastocystis* spp., with ST1 and ST3 being predominant, among asymptomatic people belonging to two small fishing villages along the Tietê river (São Paulo). According to Oishi et al. [60], *Blastocystis* spp., with a prevalence of 38.7%, were the most frequently parasites found among schoolchildren in the surrounding urban area of Curitiba. The molecular typification indicated various STs, in the order of prevalence ST3, ST1, and ST2, and a mixed infection of ST1 + ST3. de Melo et al. [57] indicated that approximately one-third of patients with diabetes mellitus in the Goias State were hosts of *Blastocystis* spp., and phylogenetic analyses revealed six STs, i.e., ST1, ST2, ST3, ST6, ST7, and ST8. Other studies also reported the great genetic variety of *Blastocystis* spp. in Brazil, indicating, at the same time, that the enteric parasites still represent a serious health concern principally due to educational deficits, poor socioeconomic rank, and inadequate sanitary conditions [55,56,62].

Current Colombian epidemiological evaluations informed eleven *Blastocystis* STs [67,68,69,70,71,72,73,74,75,76,77]. The first three STs (ST1, ST2, and ST3) were found in many demographic groups, both urban and rural [70,72,76]. Ramírez et al. [76] reported ST1, ST2, ST3, ST4, ST6, and ST7 in symptomatic (abdominal pain, anal pruritus, and diarrhea) and asymptomatic children from nine central oriental Colombian regions. The first four STs were also reported by Villamizar et al. [74], who carried out a descriptive epidemiological study on schoolchildren and their pets in Cauca (Southwest Colombia). No association was identified between *Blastocystis* spp. infection and any sociodemographic indicator; rather, the presence of STs protozoa in both humans and domestic animals suggested a zoonotic transmission. Potes-Morales et al. [73] found *Blastocystis* spp. (ST1, ST2, and ST3) when analyzing human fecal samples from Ibague. Finally, Osorio-Pulgarin et al. [70] performed a parasite molecular epidemiological analysis in a group of children (0–5 years) attending daycare centers in Medellin, and indicated that *Blastocystis* spp., with a prevalence of 15.8%, were the most frequent protozoa, followed by *Giardia* spp. and *Endolimax nana*. Additionally, six STs were identified, i.e., ST1, ST2, ST3, ST4, ST6, and the uncommon ST16. Colombia still faces numerous barriers in improving healthcare services for its citizens due to both its geography, with wide-ranging landscapes, and socioeconomic inequity [89]. Neglected tropical diseases such as *Blastocystis* negatively affect the lives of people with low incomes [90].

Reports from Argentina indicated that (1) approximately two-thirds (57.3%) of indigenous people living in the rural settlement of Puerto Iguazú (Misiones) were hosts of *Blastocystis* spp., and the parasite transmission occurred mainly through direct contact with fecal matter and contaminated water [50]; in addition, (2) one-quarter (24.8%) of patients attending the University Hospital of Cordoba City were infected with *Blastocystis* spp. [51]. In both Argentinian studies, subtypes ST1, ST2, and ST3 were found, with the latter being the most common among symptomatic and asymptomatic people [50,51].

Molecular studies, performed in rural contexts in Bolivia, showed high prevalence of intestinal parasites among children and teenagers and, at the same time, pointed out the risk of zoonotic pathogen transmission. In particular, the cross-sectional parasitological survey realized by Aruni Chura et al. [52] evidenced three *Blastocystis* spp. STs (more specifically, ST1, ST2, and ST3) among schoolchildren from ecological zones in the Department of La Paz. Macchioni et al. [53] suggested that contaminated drinking water, a lack of basic sanitary services, and close contact with animals could increase the transmission of *Blastocystis* ST2 and ST9 (isolated on very few occasions) among children living in rural settlements of the Chaco region.

Intestinal parasite infections, especially *Blastocystis* spp., are common in Latin American rural communities in Ecuador, Mexico, and Panama, as widely proven by Helenbrook, Shields, and Whipps [78], Naceanceno et al. [79], Nieves-Ramírez et al. [82], Rojas-Velázquez et al. [81], and Perea et al. [84]. The most frequent circulating *Blastocystis* STs in these groups were ST1, ST2, ST3, and ST4. The *Blastocystis* spp. prevalence rate in the Mexican general public varied from 39.6% [83] to 68.1% [81].

These results are consistent with other reports from several geographic regions worldwide that principally identified the subtypes ST1 to ST9 [91,92,93,94,95,96]. According to Nemati et al. [11], the first three, ST1, ST2, and ST3, are the most frequent STs among human subjects in the Asian continent. Karimi et al. [97] indicated that approximately 90% of the *Blastocystis* STs isolated from human fecal samples worldwide belonged to ST1, ST2, ST3, and ST4. Some studies in developing countries reported the following *Blastocystis* spp. prevalence and the dominant ST: Algeria, 7.4%—ST3 [98]; Angola, 25.6%—ST3 [99]; Azerbaijan, 45.1%—ST3 [100]; Cambodia, 55.2%—ST1 [101]; Egypt, 47.8%—ST3 [91]; India, 27.0%—ST3 [102]; Jordan, 15.0%—ST3 [100]; Malaysia, 18.5%—ST3 [103]; Nigeria, 55.5%—ST1 [100], the Philippines, 13.0%—ST3 [104]; Qatar, 71.1%—ST3 [26]; Saudi Arabia, 68.6%—ST3 [105]; Senegal, 51.7%—ST2 [106]; Sudan, 47.5%—ST1 [100]; Tanzania, 61.0%—ST1 [107]; Turkey, 24.6%—ST3 [94].

Tourists that visit Latin America or other developing countries may acquire *Blastocystis* STs during their stay, as demonstrated by van Hattem et al. [108].

As has been shown, the main routes of *Blastocystis* infection are: anthroponotic transmission, contaminated food and water, as well as close contact with animals [28,109,110,111,112,113]. The presence of *Blastocystis* spp. cysts, reported in water environments worldwide (rivers, lakes, streams, and lagoons), indicates fecal contamination of the water resources by humans or animals [11,38]. Since *Blastocystis* spp. and their STs (especially ST1 and ST3) were found in animals, including dogs, rats, cows, monkeys, and chickens, zoonotic pathways pose a serious concern for health systems in Latin American countries [74,76,114].

In spite of the fact that molecular epidemiological studies about *Blastocystis* spp. and their STs have been conducted in the last years in several Latin American countries, more studies are required to clarify the circulating STs in this continent.

### 4.2. Protocols for Blastocystis spp. Molecular Analysis

A wide range of approaches based on DNA techniques are available for detection, investigation, and surveillance of pathogenic enteroparasites [115]. PCR techniques are flexible, adaptable, and allow the automated processing of large numbers of samples in a short time [116].

The protocol to develop a descriptive study on molecular parasitology in a specific population group must be conducted under the ethical principles and approval of a bioethics committee.

The first step in carrying out a descriptive study on *Blastocystis* spp. prevalence is to establish the sample size representative of the target population. The minimum sample size (*n*) could be estimated through the following equation, as described by Oishi et al. [60]:

(1) $$n=\frac{N\alpha^{2}Z^{2}}{\left(N-1\right)e^{2}+\gamma^{2}Z^{2}}$$

where *N* is the study population, *α* is the sample standard deviation (usually set at 0.5), *Z* corresponds to the confidence level (for a 95% level of confidence, the critical value of *Z* is 1.96), *e* is the marginal error (usually set at 5%), and *γ* is the expected prevalence (usually set based on the intestinal parasite prevalence rate in close regions).

The recruited voluntary participants should be knowledgeable about the methodology and the benefits of the study, sign an informed consent form [117,118], and answer a survey that includes sociodemographic patterns (age, sex, socioeconomic data, health affiliation, and level of instruction), hygienic condition of the dwellings (sanitary services and potable water, and presence of domestic animals), and behavioral aspects (personal hygiene and hand washing, and consuming raw or half-cooked meat). Furthermore, each participant must receive basic instructions for a correct fecal sample collection in a plastic container.

The recollected stool samples are generally stored in refrigerated boxes and transported to the laboratory. Prior to the laboratory processing analysis, macroscopic examination of all stool samples evaluates their consistency and the presence of mucus [55]. Each sample could be divided into two aliquots for different uses: (1) the first aliquot is immediately frozen at −20 °C for extracting genomic DNA, and (2) the second fecal aliquot is used for parasite concentration processing (thought common techniques, such as Ritchie methods and Kato-Katz) or to direct microscopic examination. Additionally, the samples that test positive for *Blastocystis* under microscopic observation are destined for genomic analysis.

*Blastocystis* spp. DNA can be extracted from 200 mg of concentrated fecal material by manual methods or commercial kits, such as the QIAamp Stool Mini Kit (QIAGEN, Hilden, Germany) [50,55,69] and the Norgen Stool DNA Isolation Kit (Norgen Biotek Corporation, Thorold, ON, Canada) [71,72,74].

For *Blastocystis* detection and molecular subtyping, the genomic DNA is generally subjected to PCR [51,53], qPCR [75,76], or nested PCR [69,119]. A fragment of about 600 bp from the SSU rDNA gene could be amplified using the BhRDr primer combined with the RD5 primer, according to standard protocol [50,52,55].

The PCR products are usually purified with ExoSap and sequenced by the Sanger method for determining the nucleotide sequences [72]. The obtained sequences are compared to those of *Blastocystis* STs previously deposited in GenBank using the BLAST application (www.ncbi.nlm.nih.gov/BLAST, accessed on 17 January 2024). *Blastocystis* sequences could be submitted to the *Blastocystis* 18S database (http://pubmlst.org/blastocystis/, accessed on 17 January 2024) for subtype confirmation [58,60].

### 4.3. Limitations

Approximately one-third of Latin American countries have published studies on the *Blastocystis* spp. molecular epidemiology in their territories during the last nine years. The lack of data from various regions, as well as the peculiarities of each selected study and differences in populations, sampling strategies, and molecular methods (concentration, extraction, and detection) for *Blastocystis* spp. and the STs, do not allow for a more detailed analysis or the completion of a meta-analysis.

## 5. Conclusions

The data found in the selected articles indicate that the prevalence of *Blastocystis* spp. in Latin American populations has a significant variation, ranging between 5.8% and 94.0%. PCR techniques are frequently implemented to detect *Blastocystis* STs circulating in Latin America. Ten STs were reported and, in particular, the first three STs (ST1, ST2, and ST3) are widely diffused in Latin America, while ST5 and ST16 were reported in very few studies. The most recent data on *Blastocystis* spp. molecular epidemiology and STs in communities of Latin America are limited to studies from specific countries, including Argentina, Bolivia, Brazil, Chile, Colombia, Ecuador, Honduras, Mexico, Panama, and Peru. Further studies from other countries are required to obtain a complete picture and truly understand the distribution and prevalence of *Blastocystis* spp. and their STs.

## Figures and Tables

**Table 1:** General characteristics of the selected studies published.

Country	Reference	Collection Period	Group Studied	Age (Years)	Number of Repeat Samples
Argentina	Candela et al., 2021 [50]	2018	Aboriginal Communities	1–87	1
	Casero et al., 2015 [51]	NR	Patients (Urticaria)	1–57	4
Bolivia	Aruni Chura et al., 2021 [52]	2019	Schoolchildren	8–9	1
	Macchioni et al., 2016 [53]	2013	Rural Communities	1–83	NR
	Macchioni et al., 2015 [54]	2011	Children	2–12	NR
Brazil	Bertozzo et al., 2022 [55]	2018–2020	Patients (Laboratory)	0–88	NR
	Cabrine-Santos et al., 2021 [56]	2011–2012	Patients (Laboratory)	6–10	NR
	Melo et al., 2021 [57]	2015–2016	Patients (Diabetes Mellitus)	18–89	NR
	Silva et al., 2020 [58]	2011–2013	Patients (Transplant)	19–83	NR
	Melo et al., 2019 [59]	2017–2018	Patients (Urticaria)	13–73	NR
	Oishi et al., 2019 [60]	2014	Schoolchildren	0–15	NR
	Barbosa et al., 2018 [61]	2013	Agriculture Communities	2–87	1–3
	Oliveira-Arbex et al., 2018 [62]	2012–2013	Children	0–6	NR
	Seguí et al., 2018 [63]	2015–2016	General Public	0–76	NR
	Barbosa et al., 2017 [64]	NR	Patients (Psychiatric Hospital)	1–85	NR
	David et al., 2015 [65]	2011–2013	Poor Communities	2–75	3
Chile	Peña et al., 2020 [66]	2017–2018	Patients (Inflammatory Bowel Syndrome)	27–44	NR
Colombia	García-Montoya, Galván-Díaz, and Alzate, 2023 [67]	NR	Children	<5	NR
	Hernández et al., 2023 [68]	2017	Schoolchildren	4–16	1
	Hernández et al., 2021 [69]	NR	Patients (Spondyloarthritis)	18–65	1
	Osorio-Pulgarin et al., 2021 [70]	2018–2019	Children	0–5	NR
	Castañeda et al., 2020 [71]	NR	Schoolchildren	1–5	NR
	Higuera et al., 2020 [72]	NR	General Public	1–70	NR
	Potes-Morales et al., 2020 [73]	2017–2018	General Public	NR	NR
	Villamizar et al., 2019 [74]	NR	Schoolchildren	1–5	NR
	Espinosa Aranzales et al., 2018 [75]	2015–2016	Pregnant Women	14–43	1–2
	Ramírez et al., 2017 [76]	2012–2013	Children	5–14	NR
	Sánchez et al., 2017 [77]	NR	Indigenous Children	1–15	NR
Ecuador	Helenbrook et al., 2015 [78]	2011	Rural Communities	NR	1
Honduras	Naceanceno et al., 2020 [79]	NR	Children	0–13	1
Mexico	Ramírez Pérez et al., 2020 [80]	2018	University Students	19–21	3
	Rojas-Velázquez et al., 2019 [81]	2014	General Public	2–51	NR
	Nieves-Ramírez et al., 2018 [82]	2014	General Public	10–53	NR
	Rojas-Velázquez et al., 2018 [83]	2015	General Public	2–51	3
Panama	Perea et al., 2020 [84]	2017	Children	1–12	1
Perú	Ascuña-Durand et al., 2020 [85]	NR	General Public	2–82	NR

NR: not reported.

**Table 2:** Molecular characteristics of the selected studies in the systematic review.

Country	Reference	Concentration Method	DNA Extraction Method	*Blastocystis*-Specific SSU-rDNA Primers	Product Size (bp)	Amplification	STs	dST
Argentina	Candela et al., 2021 [50]	Ritchie concentration technique	QIAamp DNA Stool mini kit (QIAGEN, Hilden, Germany)	RD5 BhRDr	600	PCR	ST1 ST2 ST3	ST3
Argentina	Casero et al., 2015 [51]	Formalin-ethyl acetate Hoffman sedimentation concentration technique	QIAamp DNA Stool mini kit (QIAGEN, Hilden, Germany)	RD5 BhRDr	NR	PCR	ST1 ST2 ST3 ST6	ST3
Bolivia	Aruni Chura et al., 2021 [52]	Kato–Katz	Faecal DNA kit (Bioline, UK)	RD5 BhRDr	600	PCR	ST1 ST2 ST3	ST1
Bolivia	Macchioni et al., 2016 [53]	Ridley concentration	NucleoSpin Tissue kit (Macherey-Nagel, Duren, Germany)	F1 R1	1100	PCR	ST1 ST2 ST3	ST3
Bolivia	Macchioni et al., 2015 [54]	Ridley concentration	NucleoSpin Tissue kit (Macherey-Nagel, Duren, Germany)	F1 R1	NR	PCR	ST2 ST9	ST2
Brazil	Bertozzo et al., 2022 [55]	Centrifugation–sedimentation method	QIAamp DNA Stool mini kit (QIAGEN, Hilden, Germany)	RD5 BhRDr	600	PCR	ST1 ST2 ST3 ST4 ST6 ST7 ST9	ST3
Brazil	Cabrine-Santos et al., 2021 [56]	Ritchie concentration technique	Magnex DNA kit (Labtest Diagnóstica S.A., Minas Gerais, Brazil)	F1 R1	1100	PCR-RFLP	ST1 ST2 ST3	ST1
				SSU907 F-BH SSU907 R-BH	907			
				SSU850 F-BH SSU850 R-BH	850			
Brazil	Melo et al., 2021 [57]	Flotation zinc sulfate	QIAamp DNA Stool mini kit (QIAGEN, Hilden, Germany)	RD5 BhRDr	600	PCR	ST1 ST2 ST3 ST6 ST7 ST8	ST1
Brazil	Silva et al., 2020 [58]	Spontaneous sedimentation technique	QIAamp DNA Stool mini kit (QIAGEN, Hilden, Germany)	RD5 BhRDr	600	PCR	ST1 ST2 ST3 ST7	ST3
Brazil	Melo et al., 2019 [59]	Flotation zinc sulfate	QIAamp DNA Stool mini kit (QIAGEN, Hilden, Germany)	RD5 BhRDr	NR	PCR	ST1 ST2 ST3 ST4 ST6 ST1 + ST3	ST3
Brazil	Oishi et al., 2019 [60]	Ritchie concentration technique	QIAamp DNA Stool mini kit (QIAGEN, Hilden, Germany)	RD5 BhRDr	600	PCR	ST1 ST2 ST3 ST1 + ST3	ST3
Brazil	Barbosa et al., 2018 [61]	Spontaneous sedimentation technique Flotation-saturated sodium chloride solution Pavlova’s medium	Qiamp DNA Stool mini kit (Qiagen, Valencia, CA, USA)	Blast 505–532 Blast 998–1017	500	PCR	ST1 ST2 ST3 ST4 ST8	ST3
Brazil	Oliveira-Arbex et al., 2018 [62]	NR	QIAamp DNA Stool mini kit (QIAGEN, Hilden, Germany)	RD5 BhRDr	600	PCR	ST1 ST2 ST3 ST7	ST1
Brazil	Seguí et al., 2018 [63]	Ritchie concentration technique Kato–Katz	QIAamp DNA Stool mini kit (QIAGEN, Hilden, Germany)	RD5 BhRDr	600	PCR	ST1 ST2 ST3 ST4 ST6 ST8	ST3
Brazil	Barbosa et al., 2017 [64]	Spontaneous sedimentation technique Pavlova’s medium	Qiamp DNA Stool mini kit (Qiagen, Valencia, CA, USA)	Blast 505–532 Blast 998–1017	500	PCR	ST1 ST2 ST3 ST4 ST1 + ST3	ST3
Brazil	David et al., 2015 [65]	Flotation zinc sulfate	QIAamp DNA Stool mini kit (QIAGEN, Hilden, Germany)	RD5 BhRDr	600	PCR	ST1 ST2 ST3 ST6 ST7 ST1 + ST3 ST3 + ST7	ST3
Chile	Peña et al., 2020 [66]	PARA-PAK	QIAamp DNA Stool mini kit (QIAGEN, MD, USA)	RD5 BhRDr	600	PCR	ST1 ST2 ST3 ST4	NR
Colombia	García-Montoya, Galván-Díaz, and Alzate, 2023 [67]	Ritchie concentration technique	Norgen Stool DNA isolation kit (Norgen Biotek Corporation, Thorold, Canada)	18S-V4F	NR	PCR	ST1 ST2 ST3 ST1 +ST3	ST2
				18S-V4R				
Colombia	Hernández et al., 2023 [68]	Mini-parasep SF faecal parasite concentrator (DiaSys Ltd., Berkshire, UK)	QIAamp DNA Stool mini kit (QIAGEN, Hilden, Germany)	Blast 505–532 Blast 998–1017	500	PCR	ST1 ST2 ST3 ST4 ST5 ST1 + ST2 ST1 + ST3 ST2 + ST5 ST3 + ST5 ST1 + ST2 + ST3 ST2 + ST3 + ST4	ST2
Colombia	Hernández et al., 2021 [69]	Mini-parasep SF faecal parasite concentrator (DiaSys Ltd., Berkshire, UK)	QIAamp DNA Stool mini kit (QIAGEN, Hilden, Germany)	RD5 Blasto18SR	1722	Semi-nested PCR	ST1 ST2 ST3 ST6	ST1
				RD5 BhRDr	600	Nested PCR		
Colombia	Osorio-Pulgarin et al., 2021 [70]	Ritchie concentration technique	QIAamp DNA Stool mini kit (QIAGEN, Hilden, Germany)	FWD F5 R F2	NR	PCR	ST1 ST2 ST3 ST4 ST6 ST16	ST3
				RD5 BhRDr				
Colombia	Castañeda et al., 2020 [71]	Ritchie concentration technique Kato–Katz	Norgen Stool DNA isolation kit (Norgen Biotek Corporation, Thorold, ON, Canada)	RD5 BhRDr	600	PCR	NR	NR
Colombia	Higuera et al., 2020 [72]	NR	Norgen Stool DNA isolation kit (Norgen Biotek Corporation, Thorold, ON, Canada	RD5 BhRDr	NR	PCR	ST1 ST2 ST3 ST4 ST8 ST9	ST1
				FWD F5 R F2				
Colombia	Potes-Morales et al., 2020 [73]	Flotation zinc sulfate	Phenol-chloroform isoamyl alcohol	b11400ForC b11710RevC	310	PCR	ST1 ST2 ST3	ST1
Colombia	Villamizar et al., 2019 [74]	Ritchie concentration technique Kato–Katz	Norgen Stool DNA isolation kit (Norgen Biotek Corporation, Thorold, ON, Canada)	RD5 BhRDr	NR	PCR	ST1 ST2 ST3 ST4	ST3
Colombia	Espinosa Aranzales et al., 2018 [75]	Formol-ethyl technique	Norgen Stool DNA isolation kit (Norgen Biotek Corporation, Thorold, ON, Canada)	FWD F5 R F2	NR	qPCR	NR	NR
Colombia	Ramírez et al., 2017 [76]	NR	MP FastDNA soil kit (MP Biochemicals, Solon, OH, USA)	RD5 BhRDr	NR	qPCR	ST1 ST2 ST3 ST4 ST6 ST7	ST3
Colombia	Sánchez et al., 2017 [77]	NR	Norgen Stool DNA isolation kit (Norgen Biotek Corporation, Thorold, ON, Canada)	RD5 BhRDr	NR	qPCR	ST1 ST2 ST3 ST4 ST6	ST3
Ecuador	Helenbrook et al., 2015 [78]	NR	QIAamp DNA Stool mini kit (QIAGEN, Hilden, Germany)	BLF BLR	260	PCR	ST1 ST2 ST3	ST1
				BH1F BhRDr	600			
				b11400ForC b11710RevC	NR			
Honduras	Naceanceno et al., 2020 [79]	Kato–Katz	MP FastDNA soil kit (MP Biochemicals, Solon, OH, USA)	BL18SPPF1 BL18SR2PP	320–340	Multi-parallel qPCR	NR	NR
Mexico	Ramírez Pérez et al., 2020 [80]	Ritchie concentration technique	Commercial kit (Omega Bio-Tek Inc., Norcross, GA, USA)	F1 R1	NR	PCR	ST1 ST2 ST3 ST4 ST5 ST7	ST3
				SB82 F SB82 R	462			
				SB155 F SB155 R	650			
				SB227 F SB227 R	526			
				SB228 F SB228 R	473			
				SB229 F SB229 R	631			
				SB332 F SB332 R	338			
				SB340 F SB340 R	704			
				SB337 F SB337 R	487			
Mexico	Rojas-Velázquez et al., 2019 [81]	NR	QIAamp DNA Stool mini kit (QIAGEN, Hilden, Germany)	Blast 505–532 Blast 998–1017	NR	PCR	ST1 ST2 ST3 ST1 + ST2 ST1 + ST3 ST2 + ST3	ST3
Mexico	Nieves-Ramírez et al., 2018 [82]	NR	QIAamp DNA Stool mini kit (QIAGEN, Hilden, Germany)	RD5 BhRDr	NR	qPCR	ST3	ST3
Mexico	Rojas-Velázquez et al., 2018 [83]	NR	QIAamp DNA Stool mini kit (QIAGEN, Hilden, Germany)	RD5 BhRDr	600	PCR	ST3	ST3
Panama	Perea et al., 2020 [84]	Formalin-ethyl acetate	QIAamp DNA Stool mini kit (QIAGEN, Hilden, Germany)	BL18SPPF1 BL18SR2PP	320–340	PCR	ST1 ST3	ST1
Perú	Ascuña-Durand et al., 2020 [85]	Concentration saline solution	Norgen Stool DNA isolation kit	SB83 Sub 1 F SB83 Sub 1 R	351	PCR	ST1 ST2 ST3 ST1 + ST3 ST1 + ST2 + ST3	ST3
				STs2 F STs2 R	1500			
				SB227 Sub 3 F SB227 Sub 3 R	526			

NR: not reported; PCR: polymerase chain reaction; RT-PCR: reverse-transcription polymerase chain reaction; qPCR: quantitative polymerase chain reaction; bp: base pair; SSU-rDNA: small subunit ribosomal DNA; STs: subtypes; dST: dominant subtype.

## Data Availability

Not applicable.

## Supplementary Materials

The following supporting information can be downloaded at: https://www.mdpi.com/article/10.3390/tropicalmed9020038/s1, it contains File S1: PRISMA checklist; File S2: Quality assessment of the included studies.
