# Supplementary material for: Molecular Prevalence and Subtypes Distribution of Blastocystis spp. in Humans of Latin America: A Systematic Review
Source: Trop Med Infect Dis. 2024 Feb 1;9(2):38. doi: 10.3390/tropicalmed9020038 (PMC10893338; doi:10.3390/tropicalmed9020038)
Supplement: Supplementary file 1 [file tropicalmed-09-00038-s001.zip › Supplementary File 2.pdf]

Supplementary file 2. Quality assessment of included studies

Country	Reference	Quality Assessment Criteria Probing Questions (Q)									Study level quality score		Quality
		Q1	Q2	Q3	Q4	Q5	Q6	Q7	Q8	Q9	Total No Yes (Y)	Percentage of Yes (Y) %	
Argentina	Candela *et al.*, 2021 [50]	Y	Y	Y	Y	Y	U	Y	Y	Y	8	88.8	High
	Casero *et al.*, 2015 [51]	Y	Y	Y	Y	Y	Y	U	Y	Y	8	88.8	High
Bolivia	Aruni Chura *et al.*, 2021 [52]	Y	Y	Y	Y	Y	U	Y	Y	Y	8	88.8	High
	Macchioni *et al.*, 2016 [53]	Y	Y	Y	Y	U	Y	U	Y	U	6	66.6	Moderate
	Macchioni *et al.*, 2015 [54]	Y	Y	Y	Y	Y	U	Y	Y	Y	8	88.8	High
Brazil	Bertozzo *et al.*, 2022 [55]	Y	Y	Y	Y	Y	Y	U	Y	Y	8	88.8	High
	Cabrine-Santos *et al.*, 2021 [56]	Y	Y	Y	U	Y	U	Y	U	Y	6	66.6	Moderate
	Melo *et al.*, 2021 [57]	Y	Y	Y	Y	Y	Y	U	Y	Y	8	88.8	High
	Silva *et al.*, 2020 [58]	Y	Y	Y	Y	Y	U	Y	Y	Y	8	88.8	High

Country	Reference	Quality Assessment Criteria Probing Questions (Q)									Study level quality score		Quality
		Q1	Q2	Q3	Q4	Q5	Q6	Q7	Q8	Q9	Total No Yes (Y)	Percentage of Yes (Y) %	
	Melo *et al.*, 2019 [59]	Y	Y	Y	Y	Y	U	Y	Y	Y	8	88.8	High
	Oishi *et al.*, 2019 [60]	Y	Y	Y	Y	Y	Y	U	Y	Y	8	88.8	High
	Barbosa *et al.*, 2018 [61]	Y	Y	Y	Y	Y	U	Y	Y	Y	8	88.8	High
	Oliveira-Arbex *et al.*, 2018 [62]	Y	Y	Y	Y	Y	U	Y	Y	Y	8	88.8	High
	Seguí *et al.*, 2018 [63]	Y	Y	Y	Y	Y	Y	U	Y	Y	8	88.8	High
	Barbosa *et al.*, 2017 [64]	Y	Y	Y	Y	Y	U	Y	Y	Y	8	88.8	High
	David *et al.*, 2015 [65]	Y	Y	Y	Y	Y	Y	U	Y	Y	8	88.8	High
Chile	Peña *et al.*, 2020 [66]	Y	Y	Y	Y	Y	U	Y	Y	Y	8	88.8	High
Colombia	García-Montoya, Galván-Díaz & Alzate, 2023 [67]	Y	Y	Y	Y	Y	Y	U	Y	Y	8	88.8	High

Country	Reference	Quality Assessment Criteria Probing Questions (Q)									Study level quality score		Quality
		**Q1**	**Q2**	**Q3**	**Q4**	**Q5**	**Q6**	**Q7**	**Q8**	**Q9**	**Total No Yes (Y)**	**Percentage of Yes (Y) %**	
	Hernández *et al.*, 2023 [68]	Y	Y	Y	Y	Y	U	Y	Y	Y	8	88.8	High
	Hernández *et al.*, 2021 [69]	Y	Y	Y	Y	Y	Y	U	Y	Y	8	88.8	High
	Osorio-Pulgarin *et al.*, 2021 [70]	Y	Y	Y	Y	Y	Y	U	Y	Y	8	88.8	High
	Castañeda *et al.*, 2020 [71]	Y	Y	Y	Y	Y	Y	U	Y	Y	8	88.8	High
	Higuera *et al.*, 2020 [72]	Y	Y	Y	Y	Y	U	Y	Y	Y	8	88.8	High
	Potes-Morales *et al.*, 2020 [73]	Y	Y	Y	Y	Y	U	Y	Y	Y	8	88.8	High
	Villamizar *et al.*, 2019 [74]	Y	Y	Y	Y	Y	Y	U	Y	Y	8	88.8	High
	Espinosa Aranzales *et al.*, 2018 [75]	Y	Y	Y	Y	Y	U	Y	Y	Y	8	88.8	High
	Ramírez *et al.*, 2017 [76]	Y	Y	Y	Y	Y	Y	U	Y	Y	8	88.8	High

Country	Reference	Quality Assessment Criteria Probing Questions (Q)									Study level quality score		Quality
		Q1	Q2	Q3	Q4	Q5	Q6	Q7	Q8	Q9	Total No Yes (Y)	Percentage of Yes (Y) %	
	Sánchez *et al.*, 2017 [77]	Y	Y	Y	Y	Y	U	Y	Y	Y	8	88.8	High
Ecuador	Helenbrook *et al.*, 2015 [78]	Y	Y	Y	Y	U	Y	U	U	Y	6	66.6	Moderate
Honduras	Naceanceno *et al.*, 2020 [79]	Y	Y	Y	Y	Y	U	Y	Y	Y	8	88.8	High
Mexico	Ramírez Pérez *et al.*, 2020 [80]	Y	Y	Y	Y	Y	Y	U	Y	Y	8	88.8	High
	Rojas-Velázquez *et al.*, 2019 [81]	Y	Y	Y	Y	Y	U	Y	Y	Y	8	88.8	High
	Nieves-Ramírez *et al.*, 2018 [82]	Y	Y	Y	Y	Y	Y	U	Y	Y	8	88.8	High
	Rojas-Velázquez *et al.*, 2018 [83]	Y	Y	Y	Y	Y	U	Y	Y	Y	8	88.8	High
Panama	Perea *et al.*, 2020 [84]	Y	Y	Y	Y	Y	Y	U	Y	Y	8	88.8	High

Country	Reference	Quality Assessment Criteria Probing Questions (Q)									Study level quality score		Quality
		Q1	Q2	Q3	Q4	Q5	Q6	Q7	Q8	Q9	Total No Yes (Y)	Percentage of Yes (Y) %	
Perú	Ascuña-Durand *et al.*, 2020 [85]	Y	Y	Y	Y	Y	Y	U	Y	Y	8	88.8	High

Q1: Was the sample frame appropriate to address the target population?

Q2: Were study participants sampled in an appropriate way?

Q3: Was the sample size adequate?

Q4: Were the study subjects and the setting described in detail?

Q5: Was the data analysis conducted with sufficient coverage of the identified sample?

Q6: Were valid methods used for the identification of the condition?

Q7: Was the condition measured in a standard, reliable way for all participants?

Q8: Was there appropriate statistical analysis?

Q9: Was the response rate adequate, and if not, was the low response rate managed appropriately?
